# The Correlation between the Results of the Sniffin’ Sticks Test, Demographic Data, and Questionnaire of Olfactory Disorders in a Hungarian Population after a SARS-CoV-2 Infection

**DOI:** 10.3390/jcm12031041

**Published:** 2023-01-29

**Authors:** András Molnár, Stefani Maihoub, Panayiota Mavrogeni, Magdolna Krasznai, László Tamás, Helga Kraxner

**Affiliations:** 1 Department of Otorhinolaryngology and Head and Neck Surgery, Faculty of Medicine, Semmelweis University, Szigony u. 36, 1083 Budapest, Hungary; 2 Tóth Ilona Health Service Clinical Medical Institute, Görgey Artúr tér 8, 1212 Budapest, Hungary; 3 Department of Voice, Speech and Swallowing Therapy, Faculty of Health Sciences, Semmelweis University, Vas u. 17, 1088 Budapest, Hungary

**Keywords:** SARS-CoV-2, olfactory disorders, Sniffin’ Sticks test, quality of life, patient-reported outcome measures

## Abstract

Background: After a severe acute respiratory syndrome coronavirus-2 (SARS-CoV-2) infection, smell disorders frequently occur, significantly affecting patients’ quality of life (QoL). Methods: 110 patients with persistent olfactory disorder after coronavirus infection were enrolled. These patients underwent chemosensory testing using the Sniffin’ Sticks test, and completed the Questionnaire of Olfactory Disorders (QOD). Results: 30% of the patients reported anosmia, and 70% reported hyposmia. Upon comparing subjective and chemosensory testing categories, good category matching was observed in 75.3% (i.e., anosmia based on both methods in 10 and hyposmia in 48 cases). Statistical analysis using the Chi-square test revealed a significant result (*p* = 0.001 *). Between the TDI (i.e., Threshold, Discrimination, Identification) results of the three subjective report groups (i.e., hyposmia, anosmia, and parosmia), no significant differences were observed. When the TDI and QOD results were compared, no consistent significant correlations were found in most TDI and QOD outcomes. Between the TDI and Scale 2 results, a significant, although slight correlation was observed by the Spearman’s (*rho* = 0.213, *p* = 0.027 *) and Pearson’s (*rho* = 0.201, *p* = 0.037 *) tests. Conclusions: The nonsignificant correlation between objective and subjective methods suggests that these results should be interpreted independently. Moreover, adequate management is essential even in mild cases.

## 1. Introduction

In addition to the severe and life-threatening complications of severe acute respiratory syndrome coronavirus-2 (SARS-CoV-2) infection [1], smell and taste disorders are also important based on their significant effects on patients’ quality of life (QoL) [2]. Previous research has shown that up to 85.6% of COVID-19 patients had olfactory dysfunction (OD) related to coronavirus infection [3]. Furthermore, the prevalence of OD has been reported to be significantly higher in the case of coronavirus infection than in negative cases [4]. The loss of smell after coronavirus infection can be explained by direct damage to non-neuronal cells of the olfactory epithelium, resulting in changes in normal processing of odorants [5]. Furthermore, MRI investigations revealed a correlation between bulb size and smell loss [6]. The frequency of OD after SARS-CoV-2 infection depends on the detection method, i.e., using the standard psychophysical tests (e.g., the Sniffin’ Sticks test) or case history/subjective description of the complaints. However, there are controversial data on the suitability of objective and subjective methods in the literature. For example, a meta-analysis on olfactory loss reported a prevalence estimate of 77% vs. 44% obtained through objective and subjective methods [7]. However, it must be mentioned that this meta-analysis only included studies for objective testing on Caucasians, while studies regarding subjective methods also included investigations on Asians. Ethnic and geographic differences also influence OD, e.g., Asians present a lower prevalence of OD, probably due to different virus variants, as well as genetic background (i.e., differences in the frequency of risk alleles) [8,9,10]. According to a recent meta-analysis, 5.6% of patients report long-lasting olfactory dysfunction and 4.4% report the same for taste dysfunction, respectively [11]. Furthermore, a previous investigation stated that 12.8% of previously hospitalised COVID-19 patients still report persistent OD after one year of infection [12]. Permanent cases of OD are more relevant regarding the consequences of QoL. Therefore, there could be discrepancies between self-reported and objectively measured OD. Patient-reported outcome measures (PROMs) are essential in everyday practice; the Questionnaire of Olfactory Disorders (QOD) is an example of a questionnaire that allows the measurement of olfactory-specific QoL [13]. Concerning objective olfactory testing, it is essential to state that approximately 30% of the healthy population might have OD according to objective testing, even prior to coronavirus infection [14]. Therefore, it is necessary to determine the baseline OD test outcome prior to the pandemic; however, these results are usually inaccessible. This also suggests possible concerns regarding objective testing, which must also be considered [15]. 

Given the significant effects of OD on the patients’ QoL, this study aimed to analyse their correlations, as well as correspondences between psychophysical testing and PROMs. 

## 2. Materials and Methods

### 2.1. Participants

In this study, we enrolled 110 patients (35 men, 75 women, mean age ± SD, 37.5 years ± 11) with persistent OD. These patients were examined due to smell disorders after a SARS-CoV-2 infection in the Outpatient Clinic for Smell and Gustatory Disorders at the Department of Otorhinolaryngology and Head and Neck Surgery of Semmelweis University. The study was carried out between June 2021 and May 2022. The mean duration from the first appearance of symptoms was 12.35 ± 2.41 months, with a minimum duration of 1 month and a maximum duration of 20 months. Concerning the study period, and the appearance of SARS-CoV-2 variants (note: the first infections with the Omicron variant in Hungary were detected at the beginning of December of 2021), the investigated participants may have been infected with Alpha, Delta, and Omicron variants. However, the effect of the different variants on OD was not a part of the present investigation. The inclusion criteria were patients over 18 years of age, who were diagnosed with a SARS-CoV-2 infection using a nasopharyngeal PCR test, with an infection manifesting as smell loss. Exclusion criteria were participants with a loss of smell with aetiology other than a SARS-CoV-2 infection (e.g., head trauma, chronic sinonasal disorders, neurodegenerative diseases, and toxic damage), and patients with well-known chronic disorders that can affect QoL. Additionally, a loss of smell that began prior to COVID-19 infection was also defined as an exclusion criterion. According to the subjective classification of symptoms, anosmia was reported in 30% (*n* = 33) and hyposmia in 70% (*n* = 77) of patients. Furthermore, 56.4% of the participants also reported parosmia at the time of examination. All participants included in the present study gave their informed consent in writing. This study was approved by the Semmelweis University Regional and Institutional Committee for Science and Research Ethics (approval number: SE RKEB 65/2022).

### 2.2. Examinations

First, a detailed case history was obtained from all participants, including questions about their subjective symptoms, the first appearance of symptoms, and other diseases (e.g., internal medicine conditions, otorhinolaryngological, neurological and psychiatric diseases, and allergy), and medicines that patients took regularly. All participants underwent a detailed otorhinolaryngological examination. In addition, a brain MRI was performed for all participants, and a CT in the necessary cases (e.g., in terms of symptoms of chronic rhinosinusitis or a deviated nasal septum). As the next step in the investigation, chemosensory testing for all participants was performed using a Sniffin’ Sticks test (Burghart Messtechnik, Holm, Germany) carried out by a professional assistant [16]. An extended test was consistently applied, and at the end of the examination, the odour threshold (‘*T*’), discrimination (‘*D*’), and identification (‘*I*’) values were calculated. The test is used to differentiate between normosmia, hyposmia, and anosmia. Odour threshold testing uses n-butanol or 2-phenyl ethanol testing, and a single staircase that includes 16 triplets of odours (=48 pens). The discrimination testing includes 16 triplets of odorants, in which case a triple ‘forced choice’ is necessary. In the case of odour identification, 16 common odorants are used, and a multiple ‘forced choice’ is consistently applied. The total ‘TDI’ was calculated by summarising the subscores, with a maximum value of 48 points. The interpretation of the TDI is as follows: normosmia (TDI > 31 points), hyposmia (16 ≤ TDI < 31), and anosmia (TDI < 16).

### 2.3. Patient-Reported Outcome Measures

All participants completed the Questionnaire of Olfactory Disorders (QOD), which was translated into Hungarian using the original German and English versions by native Hungarian speakers [13,17]. This questionnaire includes 25 questions regarding olfactory-specific QoL. A patient can answer each question on a 4-point scale, with ‘agree’ (1), ’agree partly’, disagree partly’, and ‘disagree’ (4). There are 17 questions classified into negative statements that can indicate an OD-related QoL deficit in the case of a higher result score. Positive statements (2 questions) indicate a reduced ability to cope with smell loss. The questions regarding parosmia (4 questions) allowed a qualitative measurement of OD. At the end of the questionnaire, a visual analogue scale (with 5 questions) was also included, on which patients can provide an answer on a scale of 1 to 10. 

### 2.4. Statistical Analysis

Data processing was performed using the IBM SPSS Statistics for Windows, version 25 (IBM Corp., Armonk, NY, USA). Based on the Shapiro–Wilk test, the parameters did not show a normal distribution; therefore, the Mann–Whitney *U* test was used. In addition, the Chi-square test was used for categorical analysis. Furthermore, the simple linear correlation and Pearson’s and Spearman’s tests were used to detect any correlations. A *p*-value less than 0.05 was considered statistically significant. Dot curves and boxplots were included to illustrate our data.

## 3. Results

First, participants’ characteristics were analysed. Table 1 summarises the results of these data. 

**Figure 1:**
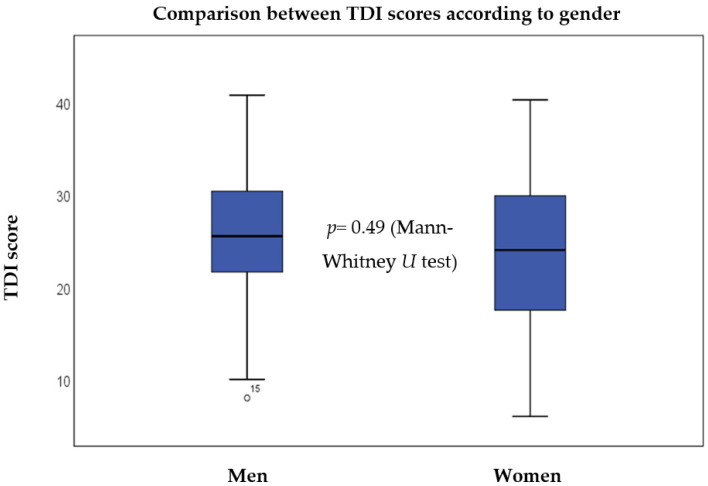
Comparison between the TDI values according to gender. The boxes represent the middle 50% of the data, and the whiskers represent the upper and lower 25%. The black line inside the boxes represents the median value. Differences were analysed using the Mann–Whitney *U* test (*p* < 0.05 *). TDI = Threshold, Discrimination, Identification.

**Figure 2:**
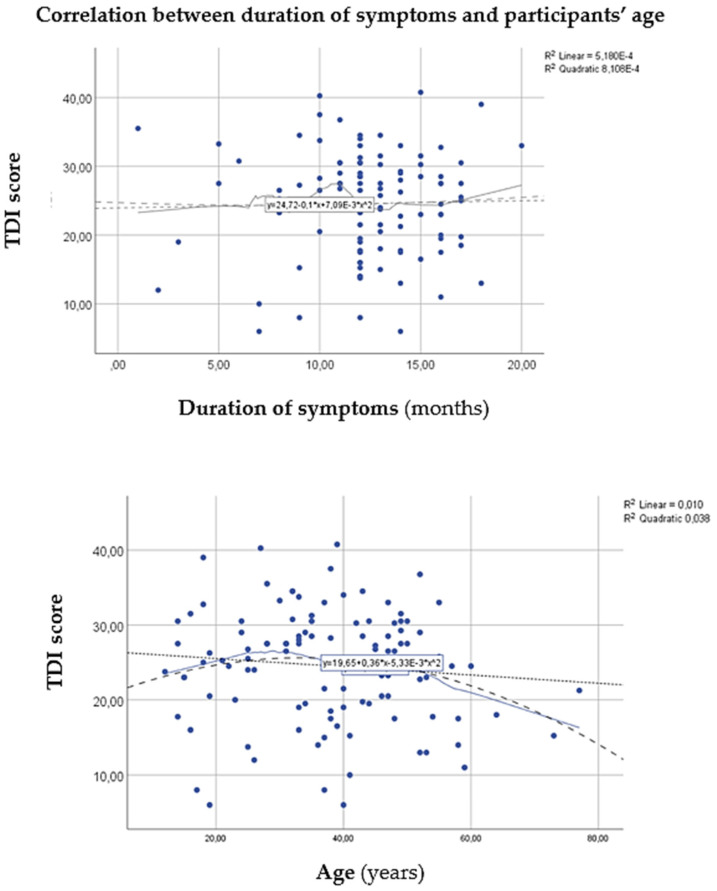
Correlation analysis between TDI and duration of symptoms and the participants’ age. Quadratic and linear regressions were used to analyse the relationship in the trends of the parameters (i.e., when age/duration is increasing, the TDI parameter is decreasing, or the contrary). The *R*^2^-values show the correlation coefficients, where a perfect correlation has an *R*^2^-value of 1. *R*^2^ = coefficient of determination; TDI = Threshold, Discrimination, Identification.

As presented in Table 1, a pronounced female predominance was observed in the basic demographic data, as more than twice as many females (i.e., 68.1%) as males were examined, with a mean age of approximately forty years. The mean duration of the complaints was 12 months. To detect a possible effect of age, gender, and the time since the first appearance of symptoms, the analyses presented in Figure 1 and Figure 2 were carried out. When data on subjective symptoms were collected, 70% of participants reported hyposmia and 30% reported anosmia. In addition, 56.4% of the patients also reported parosmia. 

As illustrated in Figure 1, there was no apparent difference between the TDI values of the two groups. Statistical analysis using the Mann–Whitney *U* test did not indicate a significant difference (*z*-score: −0.688, *p* = 0.49) between the two groups. Consequently, it can be stated that gender does not significantly affect the results of chemosensory testing. 

As Figure 2 reveals, no linear correlation was observed between the TDI score and the duration of symptoms, and the participants’ age. The Spearman’s test also did not indicate a significant nonlinear correlation between TDI and duration of symptoms (*rho* = 0.049, *p* = 0.621), and age (*rho* = 102, *p* = 0.292), respectively. Therefore, it can be concluded that the time since the first appearance of symptoms and participants’ age also did not significantly affect the objective test results. 

When the results of the chemosensory testing were compared with subjective reports, a slight difference was observed. As Table 1 presents, according to chemosensory testing, 15.5% were in the normosmia group, and anosmia was less frequent (13.5%). Consequently, the categories of subjective reports and chemosensory testing were compared using the Chi-square test, and the results are presented in Table 2. 

As shown in Table 2, the best matching was observed in the case of the hyposmia category. In some cases, patients subjectively reported anosmia, while chemosensory testing classified them into the hyposmia category. A category changing between hyposmia (subjective) and anosmia (chemosensory testing) was uncommon. Statistical analysis using the Chi-square test indicated a *p*-value of 0.001 *, indicating a significant result. However, it can be stated that the correlation between subjective complaints and chemosensory testing remains controversial.

**Figure 3:**
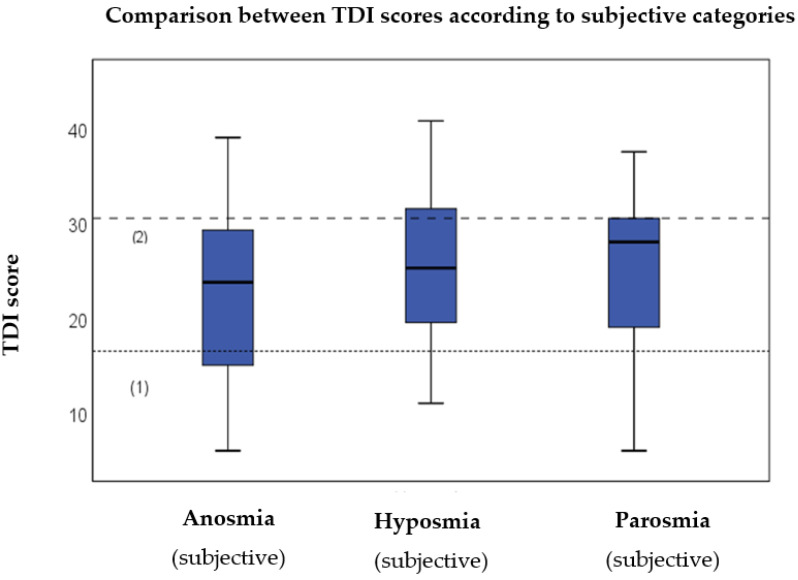
Comparison between TDI scores and subjective categories reported by the patients. (1): Reference line for anosmia (showing 16). (2): Reference line for normosmia (showing 31). The boxes represent the middle 50% of the data and the whiskers the upper and lower 25%. The black line inside the boxes represents the median value. Differences were analysed using the Mann–Whitney *U* test (*p* < 0.05 *). TDI = Threshold, Discrimination, Identification.

As can be observed from Figure 3, there is no apparent difference between the data of the three groups. When statistical analysis using the Mann–Whitney *U* test was performed, no significant differences were observed between the anosmia vs. hyposmia (*p* = 0.25), anosmia vs. parosmia (*p* = 0.17), and hyposmia vs. parosmia (*p* = 0.5) groups, respectively. These results indicate that the severity of smell loss based on chemosensory testing does not reflect the subjective categories reported by the patients or the presence of parosmia. 

As a next step in the investigation, the correlation between TDI and QOD results was analysed. Therefore, the Spearman’s and Pearson’s correlation tests were used, and the results are presented in Table 3. 

From Table 3. it can be observed that in most cases, no significant correlation between chemosensory testing (i.e., TDI according to Sniffin’ Sticks test) and QOD was found. Only in the case of the TDI score and the Scale 2 scores was a significant, although slight, correlation observed. Consequently, it can be stated that no significant correlations were detected between PROMs and chemosensory testing. Therefore, objective testing results do not reflect the severity of subjective complaints; consequently, both must be considered in terms of therapy planning.

## 4. Discussion

A total of 110 participants suffering from OD after a SARS-CoV-2 infection was included in the present investigation. A pronounced female predominance was observed in the current population, analogous to previous research results. Interestingly, this gender difference was also quite frequent in other studies; the percentages of female participants were as follows: 62.5% [18], 54.4% [19], 59% [20] or 68.1% [21], respectively. Based on previous studies, increasing age had significant effects on OD; however, in the present investigation, such a correlation was not detected [22]. OD is important due to its effect on QoL and day-to-day functioning; moreover, a recent study observed a correlation between reduced cognitive functions and hyposmia [23]. Furthermore, smell loss can be considered a public health problem [24], as many patients report short-term/persistent OD post infection (e.g., 31.8% [25], 61.7% [26] or 70.1% [27], respectively). However, it should be mentioned that most cases of COVID-related OD (i.e., approximately 95%) are short term [11]. For example, a review stated that most COVID-related OD cases resolve in a period of a few weeks [24]. A review concluded that only 20% of patients still report olfactory impairment after one month [28]. Another investigation found a prevalence of OD at 11.7% with long-term follow-up (i.e., 2 years) [29]. Furthermore, previous research has shown a correspondence between OD and depression [30]; therefore, psychiatric comorbidities could also be observed. 

Chemosensory testing, for example, using the Sniffin’ Sticks test, is essential in the objective diagnosis and follow-up of OD. However, there might be a dissociation between the results of objective measurements and subjective methods (i.e., PROMs). Although the results of the chemosensory testing have been analysed in several investigations, the prevalence of OD based on subjective reports has been particularly often investigated. A study that examined long-lasting OD after a SARS-CoV-2 infection found that, according to the Sniffin’ Sticks test, 72.5% of patients were hyposmic, 23.5% normosmic, and 4% anosmic [31]. This distribution is analogous to the results of the current investigation; however, normosmic patients based on TDI were not identified. However, other investigations showed that anosmia is more common after coronavirus infection than hyposmia [32,33]. This is in contradiction to the results of the current study. The parosmia symptom was reported, e.g., as 10.8% [34], 64.1% [35], and 89.8% [36] according to previous research results. For this symptom, the distributions are highly variable; in our study, approximately half of the study group also reported parosmia. It was reported that significantly better quantitative olfactory scores (i.e., Brief Smell Identification Test) and worse QoL were observed in the case of parosmia symptom [34]; however, such a correlation was not detected in the current investigation. An additional interesting aspect is that several studies concluded that in the case of COVID-19-related OD, the odour threshold is more affected than odour discrimination and identification scores [21,28]. This is consistent with the hypothesis that coronavirus causes OD related to the olfactory epithelium, and odorant-metabolising enzymes, rather than based on the processing in the central nervous system [10]. 

Smell disorders can significantly affect the patients’ QoL, which was also investigated in previous reports. For example, a study observed a significant correlation between smell loss and the Patient Health Questionnaire, and QOD. Furthermore, a reduced OD specific QoL was also correlated with the presence of depressive symptoms [30]. Another investigation stated that 96% of participants with COVID-19-associated smell loss reported at least one QoL deficit, while 75% reported three deficits, and 43% showed depression. In addition, ‘reduced food enjoyment’ was the most common complaint, significantly affecting patients’ QoL and day-to-day functioning [37]. Another investigation also concluded the significant effect of OD on the QoL, with 76% involvement; moreover, it also highlighted the importance of emotion-focused and coping strategies in the case of OD [38]. A systematic review found a 67.7% QoL deficit in the case of COVID-19, which can be considered as a pretty high prevalence [39]. Therefore, the use of a smell disorder-specific questionnaire, e.g., the QOD [12], which has been validated in different languages [17,40,41], is of great importance. The questionnaire has yet to be validated in the Hungarian language; this is a limitation of the current investigation. 

An additional interesting issue is a possible correlation between subjective complaints and objective testing results. Answering this question was one of the essential parts of the current investigation. The present study did not observe a consistent correlation between PROMs and objective testing (i.e., the TDI score based on the Sniffin’ Sticks test). For example, according to a systematic review, 19% was the lowest reported prevalence of OD when psychophysical testing was performed. In some cases, objective testing revealed a higher prevalence of OD, while others concluded the opposite, i.e., subjective methods detected a higher prevalence of OD [15]. These results suggest conflicting data in the literature on the sensitivities of objective and subjective methods in OD. Concerning this issue, some previous studies concluded that objective testing is more sensitive than subjective methods. 

Another investigation observed an underestimation of subjective OD compared to objective testing [42]. This result highlights the importance of olfactory testing, not only considering patients’ subjective reports. Consistently, another investigation also presented better results using objective assessment. This means that 96.6% of those who self-reported, and 72.8% of those who did not report a loss of smell, were identified as having smell loss, according to objective testing (i.e., Chemosensory Clinical Research Center test). Although, in that study, an OD-specific questionnaire was not applied, the authors only asked whether or not the participants had smell loss [43]. Remarkably similar to this, although also using questionnaires, another study also detected higher rates of smell disorders in COVID-19 patients using the Sniffin’ Sticks test. When the correlations between the results of the questionnaire and objective testing were analysed, it was reported that the objective tests detected OD at a much higher rate than the questionnaires (83.2% vs. 52.5%, respectively) [44]. Another analysis, applying the Q-SIT, also found objective methods a more suitable tool to assess OD, which can also be helpful in paucisymptomatic cases (i.e., at the early stage of the disease with less pronounced symptoms) [19]. 

In contradiction, other investigations stated that subjective methods are more sensitive than objective testing; for example, using a yes/no question and a scale of 1 to 10 [18], or an online self-administered questionnaire [45]. Additionally, other studies did not observe a significant correlation between objective and subjective methods, referring to the result that only 62% [46], 74% [18], 76% [20], and 94% [21] of patients with self-reported smell loss also presents a loss of smell based on objective psychophysical testing [46]. On the contrary, a previous investigation observed a significant, although moderate correlation between self-reported complaints and TDI [31]. Another study revealed a high concordance between self-reported and clinically observed OD [47]. This is analogous to the results of the present investigation, as no consistent correlation was revealed between TDI and QOD. Some facts can explain the lack of correlation between objective and subjective methods. First, participants are often (i.e., up to 30% of cases) unaware of their OD; therefore, subjectively, they will report a normal sense of smell, but objective testing will identify the OD (i.e., they can be classified into a false negative group) [14]. In addition, there are participants with excellent smell ability, resulting in lower thresholds and higher smell sensibility. In the case of an OD, subjectively, these participants will complain about a changed perception of smell (i.e., compared to their own baseline smell), even though this might not be revealed by objective testing. Consequently, it can be concluded that conflicting data in the literature regarding subjective reports and objective testing can be found; therefore, more research is needed on this topic. It is also important to mention that the lack of a correlation between objective and subjective methods precedes the pandemic area. Accordingly, an investigation published in 2003 did not observe a correlation between subjective (i.e., visual analogue scale) and objective (i.e., Sniffin’ Sticks test) methods [48]. 

During the COVID-19 pandemic, many investigations using online surveys were carried out [49,50,51,52]. The advantage of these surveys is that large sample size can be investigated; in addition, participants can report their symptoms without a long waiting period. However, according to the results of the current investigation, the possible utility of these online questionnaires for the diagnosis or treatment of OD is controversial. Based on our results, i.e., the discrepancy between objective testing results and subjective reports, the use of questionnaires provides an incomplete account of olfactory dysfunction. 

## 5. Conclusions

The present investigation compared objective olfactory testing and PROMs. Our results did not indicate a significant correlation between subjective findings; therefore, both must be considered during a medical consultation. The TDI scores did not differ between those who reported hyposmia, anosmia, or parosmia subjectively. Furthermore, no significant correlation was consistently observed between QoL based on QOD and objective testing results; therefore, an OD can reduce QoL independently of its severity. Consequently, adequate therapy is also essential in mild cases of smell disorders. The difference between objective and subjective methods could be explained by the fact that participants in the normal group may also have OD, which can be determined using objective methods. Furthermore, participants with excellent smell function before infection can subjectively place themselves in the impaired smell category, although objective testing will classify them into normal olfaction. 

## Figures and Tables

**Table 1:** Participants’ characteristics.

Category	Values
**Age** (mean ± SD years)	37.5 ± 11
**Gender** (men/women)	35/75
**Duration since first appearance of symptoms** (mean ± SD months)	12.35 ± 2.41
**Subjective complaint report**	
Anosmia, *n* (%)	33 (30%)
Hyposmia, *n* (%)	77 (70%)
Parosmia, *n* (%)	61 (56.4%)
**TDI results**	
Normosmia, *n* (%)	17 (15.5%)
Hyposmia, *n* (%)	78 (71%)
Anosmia, *n* (%)	15 (13.5%)

*n* = number of cases; SD = standard deviation; TDI = Threshold, Discrimination, Identification.

**Table 2:** Comparison between subjective and chemosensory testing categories. Statistical analysis was performed using the Chi-square test. A *p*-value below 0.05 was defined as a significant correlation indicated by the * sign. The parameters show the number of cases.

	**Anosmia** (Chemosensory Testing)	**Hyposmia** (Chemosensory Testing)
**Anosmia** (subjective category)	10	17
**Hyposmia** (subjective category)	4	48
	*p* = 0.001 *	

**Table 3:** Correlation analyses between the TDI and QOD results. Spearman’s and Pearson’s correlation tests were used to detect a nonlinear and linear correlation, respectively. A statistically significant correlation was defined with a *p*-value < 0.05 *. A perfect correlation can be indicated by a *rho* of 1. The * indicates a statistically significant correlation. QOD = questionnaire of olfactory disorders; TDI = Threshold, Discrimination, Identification.

	Spearman’s Test	Pearson’s Test
**TDI vs. Life Quality**	*rho* = 0.1, *p* = 0.305	*rho* = 0.040, *p* = 0.680
**TDI vs. Positive Life Quality**	*rho* = 0.040, *p* = 0.685	*rho* = 0.037, *p* = 0.702
**TDI vs. Sincerity Statement**	*rho* = 0.171, *p* = 0.133	*rho* = 0.151, *p* = 0.119
**TDI vs. Sincerity Inverse**	*rho* = 0.061, *p* = 0.532	*rho* = 0.1, *p* = 0.306
**TDI vs. Parosmia**	*rho* = 0.007, *p* = 0.940	*rho* = 0.014, *p* = 0.883
**TDI vs. Scale 1**	*rho* = 0.160, *p* = 0.098	*rho* = 0.150, *p* = 0.121
**TDI vs. Scale 2**	*rho* = 0.213, *p* = 0.027 *	*rho* = 0.201, *p* = 0.037 *
**TDI vs. Scale 3**	*rho* = 0.154, *p* = 0.112	*rho* = 0.144, *p* = 0.137
**TDI vs. Scale 4**	*rho* = 0.028, *p* = 0.776	*rho* = 0.044, *p* = 0.652
**TDI vs. Scale 5**	*rho* = 0.093, *p* = 0.340	*rho* = 0.064, *p* = 0.514

## Data Availability

The data used and/or analysed during the current study are available from the corresponding author on reasonable request.
